# The Joint Effect of *hOGG1*, APE1, and ADPRT Polymorphisms and Cooking Oil Fumes on the Risk of Lung Adenocarcinoma in Chinese Non-Smoking Females

**DOI:** 10.1371/journal.pone.0071157

**Published:** 2013-08-12

**Authors:** Xiaoxia Xue, Zhihua Yin, Yao Lu, Haibo Zhang, Ying Yan, Yuxia Zhao, Xuelian Li, Zeshi Cui, Miao Yu, Lu Yao, Baosen Zhou

**Affiliations:** 1 The Third Center of Laboratory Technology and Experimental Medicine, China Medical University, Shenyang, PR China; 2 Department of Epidemiology, School of Public Health, China Medical University, Shenyang, PR China; 3 Department of Radiotherapy, Shenyang Northern Hospital, Shenyang, PR China; 4 Department of Radiation Oncology, First Affiliated Hospital of China Medical University, Shenyang, PR China; MOE Key Laboratory of Environment and Health, School of Public Health, Tongji Medical College, Huazhong University of Science and Technology, China

## Abstract

**Background:**

The human 8-oxoguanine DNA glycosylase 1 (*hOGG1*), apurinic/apyrimidinic endonuclease 1 (APE1), and adenosine diphosphate ribosyl transferase (ADPRT) genes play an important role in the DNA base excision repair pathway. Single nucleotide polymorphisms (SNPs) in critical genes are suspected to be associated with the risk of lung cancer. This study aimed to identify the association between the polymorphisms of *hOGG1* Ser326Cys, APE1 Asp148Glu, and ADPRT Val762Ala, and the risk of lung adenocarcinoma in the non-smoking female population, and investigated the interaction between genetic polymorphisms and environmental exposure in lung adenocarcinoma.

**Methods:**

We performed a hospital-based case-control study, including 410 lung adenocarcinoma patients and 410 cancer-free hospital control subjects who were matched for age. Each case and control was interviewed to collect information by well-trained interviewers. A total of 10 ml of venous blood was collected for genotype testing. Three polymorphisms were analyzed by the polymerase chain reaction-restriction fragment length polymorphism technique.

**Results:**

We found that individuals who were homozygous for the variant *hOGG1* 326Cys/Cys showed a significantly increased risk of lung adenocarcinoma (OR = 1.54; 95% CI: 1.01–2.36; P = 0.045). When the combined effect of variant alleles was analyzed, we found an increased OR of 1.89 (95% CI: 1.24–2.88, P = 0.003) for lung adenocarcinoma individuals with more than one homozygous variant allele. In stratified analyses, we found that the OR for the gene-environment interaction between Ser/Cys and Cys/Cys genotypes of *hOGG1* codon 326 and cooking oil fumes for the risk of lung adenocarcinoma was 1.37 (95% CI: 0.77–2.44; P = 0.279) and 2.79 (95% CI: 1.50–5.18; P = 0.001), respectively.

**Conclusions:**

The *hOGG1* Ser326Cys polymorphism might be associated with the risk of lung adenocarcinoma in Chinese non-smoking females. Furthermore, there is a significant gene-environment association between cooking oil fumes and *hOGG1* 326 Cys/Cys genotype in lung adenocarcinoma among female non-smokers.

## Introduction

Lung cancer has become a major cause of cancer mortality worldwide, especially in China, and pathological studies have found that adenocarcinoma is the main form of lung cancer in the female population [1], [2]. It is well known that smoking is the most important risk factor for lung cancer, but in the past 30 years, the incidence and death rate of lung cancer is increasing in women who have a low rate of smoking [3]–[5]. Lung cancer maybe caused mainly by the other factors in Chinese women, therefore, it is important to study the factors that affect female lung cancer, especially non-smoking females.

Environmental exposure and genetic polymorphisms might contribute to the variation in individual lung cancer risk. Recent lung cancer studies have focused on identifying effects of single nucleotide polymorphisms (SNPs) in candidate genes; in particular, DNA repair genes are being increasingly studied. DNA repair systems play a fundamental role in the maintenance of genomic integrity and protect the human genome from damage caused by environmental carcinogens. Genetic variations in DNA repair genes are thought to affect DNA repair capacity and are suggested to be associated with a risk for lung cancer [6]–[8].

As one of the DNA repair pathways, the DNA base excision repair (BER) pathway plays an important role in repairing DNA damage caused by oxidation, deamination, and alkylation, and protecting cells against the damage of endogenous and exogenous carcinogens [9]–[11]. The human 8-oxoguanine DNA glycosylase 1 (*hOGG1*), apurinic/apyrimidinic endonuclease 1 (APE1) and adenosine diphosphate ribosyl transferase (ADPRT) genes play an important role in the BER pathway [12]–[14]. The *hOGG1* gene encodes a DNA glycosylase and completes repair by releasing the 8-oxoG base caused by DNA reactive oxygen [15]–[17]. The APE1 gene is located on chromosome 14q11.2-q12 and a rate -limiting enzyme in the BER pathway that repairs basic sites in DNA and functions as a redox activator, which regulates some transcription factors, and then participates in the BER process [18]–[20]. The ADPRT gene is located at chromosome 1q41–q42 region, plays a direct and important role in the long-path BER pathway and it can bind to single-strand breaks in DNA [21]–[23]. *hOGG1* Ser326Cys, APE1 Asp148Glu and ADPRT Val762Ala are three common candidate single-nucleotide polymorphisms, and there were lots of studies investigated the association between BER SNPs and the risk of cancer [24], [25]. Recently, several studies have demonstrated the associations between these genetic polymorphisms and the risk of lung cancer, but the results vary in different ethnic populations and with different environmental exposure [26]–[33].

In the present study, we performed a case-control study to identify the association between the polymorphisms of *hOGG1*, APE1, and ADPRT, and the risk of lung adenocarcinoma in the non-smoking female population in Shenyang, China. We investigated the joint effects of three genes in the same pathway and the interaction between genetic polymorphisms and environmental exposure in lung adenocarcinoma.

## Methods

### Subjects

This hospital-based case-control study included 410 lung adenocarcinoma patients and 410 cancer-free hospital controls. All subjects were non-smoking females and they were from unrelated ethnic Han Chinese. The cases were recruited during January 2002 to January 2008 at the First Affiliated Hospital of China Medical University and Shenyang Northern Hospital. All patients were histologically confirmed to have lung adenocarcinoma before any radiotherapy and chemotherapy. During the same time, controls were selected from cancer-free patients with other lung diseases, but who were free of a history of cancer, and mainly suffered from bronchitis, pulmonary disease and emphysema. Controls were frequency-matched to case subjects for age (±5 years). This study was approved by the institutional review board of China Medical University and written informed consent was obtained from all participants.

### Data Collection

A total of 10 ml of venous blood was collected from each patient. Patients were interviewed to collect information for demographics and environmental exposure by well-trained interviewers at the time they were admitted to hospital. Information included demographic characteristics, dietary habit, and family history of cancer, fuel smoke exposure, passive smoking, cooking oil fumes exposure, and occupational exposure. An individual was defined as a smoker if she had consumed a total of 100 cigarettes in her lifetime; otherwise, she was considered as a non-smoker. For exposure to cooking oil fumes, participants were asked about the frequency of cooking and types of oils.

### Genotyping Methods

Genomic DNA was extracted from peripheral blood samples using the guanidine hydrochloride (GuHCl) method. Genotyping of *hOGG1* Ser326Cys, APE1 Asp148Glu and ADPRT Val762Ala was performed using the polymerase chain reaction-restriction fragment length polymorphism (PCR-RFLP) technique. The PCR primers used were as follows: *hOGG1* Ser326Cys: 5′-GTGGATTCTCATTG CCTTCG-3′ (forward) and 5′-CTGTTGCTGTCGAGACTGC-3′ (reverse); APE1 Asp148Glu: 5′-TAATTCTGTTTCATTTCTATAGGCTA-3′ (forward) and 5′-TGCA TTAGGTACATATGCTGTT-3′ (reverse); and ADPRT Val762Ala: 5′-TTTGCTCC TCCAGGCCAACG-3′ (forward) and 5′-TGGAAGTTGGGACCGCTGC-3′ (reverse). The PCR products were digested with restriction enzyme Fnu4HI (for *hOGG1* Ser326Cys), FspBI (for APE1 Asp148Glu) and BstUI (for ADPRT Val762Ala) to determine the genotypes. More than 10% of the samples were randomly selected for repeat tests, and the results were 100% concordant.

### Statistical Analysis

We used the Pearson’s chi square test and/or student’s t-test to compare the differences in demographic variables, environmental risk factors, and genotypes of the three genes between cases and controls. The odds ratio (OR) and 95% confidence interval (95% CI) for estimating the associations between genotypes of these genes and lung cancer were used in unconditional logistic regression analysis. The Hardy-Weinberg equilibrium was tested by performing a goodness-of-fit X^2^ test to compare the genotype frequencies of each SNP in the control subjects from those expected. A logistic regression model was used to evaluate gene-gene and gene-environment interactions. All data were analyzed with Statistical Product and Service Solutions (SPSS) v13.0 for Windows, if not otherwise specified. All statistical analyses were two-sided and the significance level was set at p<0.05.

## Results

The basic demographic data and environmental risk factors of the 410 lung cancer patients and 410 controls are shown in Table 1. The mean ages of cases and controls (mean ± S.D.) were similar. All cases were female lung adenocarcinoma patients. We found no significant differences in age, passive smoking, family history of cancer, fuel smoke exposure, education, and monthly income between the case and control groups. However, more cases were exposed to cooking oil fumes than in the controls (OR 1.62; 95% CI: 1.21–2.18; P = 0.001).

**Table 1:** Basic demographic data and environmental risk factor in cases and controls.

Variable	Cases n (%)	Control n (%)	P value
Female	410	410	
Age (years)	53.05±4.48	53.61±4.13	0.063^a^
Income(yuan/month)	702.72±304.09	680.66±255.19	0.261^a^
Education			0.753^b^
Never	41 (10.0)	39 (9.5)	
Elementary school	185 (45.1)	197 (48.0)	
Junior school	128 (31.2)	115 (28.0)	
Senior school and upwards	56 (13.7)	59 (14.4)	
Family history of cancer	57 (13.9)	42 (10.2)	0.108^b^
Passive smoking	256 (62.4)	240 (58.5)	0.253^b^
Fuel smoke exposure	118 (28.8)	115 (28.0)	0.816^b^
Cooking oil fume exposure	153 (37.3)	110 (26.8)	0.001^b^ *

* P<0.05.

a Student’s t-test was used to compare the frequency distributions of demographic variables between the cases and controls.

b Pearson’s chi square was used to compare the frequency distributions of demographic variables, passive smoking, famili history of cancer, fuel smoke exposure, cooking oil fume exposure between the cases and controls.

The distribution of *hOGG1*, APE1 and ADPRT gene polymorphisms in cases and controls, and the adjusted ORs associated with lung adenocarcinoma are presented in Table 2. In the controls, all the genotype distributions were in agreement with the Hardy-Weinberg equilibrium (P>0.05). Using subjects with the *hOGG1* Ser/Ser genotype as the reference group, homozygous carriers of the *hOGG1* 326Cys/Cys genotype had a 1.54-fold risk of lung adenocarcinoma compared with the homozygous wild genotypes (95% CI: 1.01–2.36, P = 0.045). No significant association was found between APE1 148Asp/Glu polymorphisms and the risk for lung adenocarcinoma (P>0.05). Distribution of ADPRT Val762Ala polymorphisms was not associated with a risk of lung adenocarcinoma (P>0.05).

**Table 2:** Distribution of genotypes and ORs for lung adenocarcinoma cases and controls.

Genotype	Cases n (%)	Controls n (%)	OR [95%CI]^a^	P value
*hOGG1* Ser326Cys				
Ser/Ser	55 (13.4)	68 (16.6)	1.00 (reference)	–
Ser/Cys	178 (43.4)	200 (48.8)	1.10 [0.73–1.66]	0.656
Cys/Cys	177 (43.2)	142 (34.6)	1.54 [1.01–2.36]	0.045*
APE1 Asp148Glu				
Asp/Asp	116 (28.3)	130 (31.7)	1.00 (reference)	–
Asp/Glu	183 (44.6)	190 (46.3)	1.17 [0.84–1.63]	0.347
Glu/Glu	111 (27.1)	90 (22.0)	1.35 [0.93–1.97]	0.119
ADPRT Val762Ala				
Val/Val	129 (31.5)	138 (33.7)	1.00 (reference)	–
Val/Ala	202 (49.3)	205 (50.0)	1.04 [0.76–1.42]	0.794
Ala/Ala	79 (19.3)	67 (16.3)	1.29 [0.86–1.94]	0.218

a Adjusted for age, cooking oil fume and data were calculated by unconditional logistic regression.

* P<0.05.

The effect of the combination of *hOGG1* 326Cys, APE1 148Glu and ADPRT 762Ala polymorphisms on the risk of lung adenocarcinoma is shown in Table 3. The reference group was individuals who were not homozygous for at least one of the variant alleles, considering the rarity of individuals with all three alleles. When patients homozygous for two or three gene variants were combined as one group, we found an increased OR of 1.89 (95% CI: 1.24–2.88; P = 0.003) for lung adenocarcinoma individuals with more than one homozygous variant allele of *hOGG1* 326 Cys/Cys, APE1 148Glu/Glu and ADPRT 762Ala/Ala. However, no significant effect was found in lung adenocarcinoma individuals with one homozygous variant allele.

**Table 3:** The combination of *hOGG1* 326Cys, APE1 148Glu and ADPRT 762Ala polymorphisms on lung adenocarcinoma risk in individuals homozygous for more than one variant “risk allele.”

Homozygous variant allelesper individual	Cases n (%)	Controls n (%)	OR [95%CI]^a^	P value
All cases analyzed	410 (100.0)	410 (100.0)		
0	133 (32.4)	167 (40.7)	1.00 (reference)	–
1	195 (47.6)	191 (46.6)	1.29 [0.94–1.75]	0.104
2	75 (18.3)	49 (12.0)	1.83 [1.19–2.82]	0.006*
3	7 (1.7)	3 (0.7)	2.88 [0.72–11.49]	0.134
2+3	82 (20.0)	52 (12.7)	1.89 [1.24–2.88]	0.003*

a Adjusted for age and cooking oil fume, ORs and 95%CIs were calculated by logistic regression.

* P<0.05.

To evaluate the potential gene-environment interaction, the association between the polymorphisms of *hOGG1* Ser326Cys and exposure to cooking oil fumes was tested in a logistic regression model. The adjusted ORs for the interaction between *hOGG1* Ser326Cys and cooking oil fumes for the risk of lung adenocarcinoma are shown in Table 4. In order to see the direct joint effect of cooking oil fumes and hOGG1 Ser326Cys, which means to create a joint effect variable with 6 values according to exposure to cooking oil fumes (no/yes) and the SNP (Ser/Ser, Ser/Cys, Cys/Cys) and then compared to no exposure and ser/ser calculate ORs for the other 5 groups, we found that the ORs for the gene-environment interaction between Ser/Cys and Cys/Cys genotypes of *hOGG1* codon 326 and cooking oil fumes for the risk of lung adenocarcinoma was 1.37 (95% CI: 0.77–2.44; P = 0.279) and 2.79 (95% CI: 1.50–5.18; P = 0.001), respectively. In stratified analyses by passive smoking and fuel smoke exposure, we found no statistically significant relationships between the risk of lung adenocarcinoma and the three genetic polymorphisms.

**Table 4:** Association between *hOGG1* Ser326Cys polymorphisms and risk of lung adenocarcinoma stratified by cooking oil fume exposures.

Exposure to cooking oil fumes (no/yes)and the SNP (Ser/Ser, Ser/Cys, Cys/Cys)	Cases n (%)	Controls n (%)	OR [95%CI]^a^	P value
All cases analyzed	410 (100.0)	410 (100.0)		
1^b^	35 (8.5)	44 (10.7)	1.00 (reference)	–
2^c^	114 (27.8)	144 (35.1)	0.97 [0.59–1.62]	0.916
3^d^	108 (26.3)	112 (27.3)	1.17 [0.70–1.97]	0.552
4^e^	20 (4.9)	24 (5.9)	1.03 [0.49–2.16]	0.938
5^f^	64 (15.6)	56 (13.7)	1.37 [0.77–2.44]	0.279
6^g^	69 (16.8)	30 (7.3)	2.79 [1.50–5.18]	0.001*

a Adjusted for age, ORs and 95%CIs were calculated by logistic regression.

* P<0.05.

b was defined non-exposure to cooking oil fumes and Ser/Ser genotypes of *hOGG1* codon 326;

c was defined non-exposure to cooking oil fumes and Ser/Cys genotypes of *hOGG1* codon 326;

d was defined non-exposure to cooking oil fumes and Cys/Cys genotypes of *hOGG1* codon 326;

e was defined exposure to cooking oil fumes and Ser/Ser genotypes of *hOGG1* codon 326;

f was defined exposure to cooking oil fumes and Ser/Cys genotypes of *hOGG1* codon 326;

g was defined exposure to cooking oil fumes and Cys/Cys genotypes of *hOGG1* codon 326.

## Discussion

The incidence of lung cancer in non-smoking female population may be caused by genetic factors and other important environmental besides tobacco smoking, because of Chinese traditional cooking style. Cooking oil fumes are one of the major indoor air pollutants and are an important risk factor of lung cancer [34], [35]. However, the joint effect of these three genes and cooking oil fumes on the risk of lung adenocarcinoma in Chinese non-smoking females were not yet reported. Therefore, we investigated the association of three genetic polymorphisms (*hOGG1* Ser326Cys, APE1 Asp148Glu, and ADPRT Val762Ala) in BER genes with the risk of lung adenocarcinoma, as well as the relationship of the three genetic polymorphisms with environmental factors (cooking oil fumes) in the Chinese non-smoking female population. We found a significant association between the *hOGG1* 326Cys/Cys genotype and lung adenocarcinoma. Homozygous carriers of the *hOGG1* 326Cys/Cys genotype had a 1.54-fold risk of lung adenocarcinoma compared with the homozygous wild genotypes (Table 2).

Several studies have investigated the association of *hOGG1* Ser326Cys polymorphisms with lung cancer [26]–[28], [36]–[43]. Kohno et al [26] first identified *hOGG1* Ser326Cys polymorphisms and found that the 326Cys protein has a low oh8G repair activity. Our recent meta-analysis of *hOGG1* Ser326Cys using data from 18 studies showed that *hOGG1* Ser326Cys polymorphisms might contribute to the risk of non-small cell lung cancer in the Asian population [27]. Other previous studies have shown a significant positive association between the homozygous variant Cys/Cys genotype and lung cancer development, and some of these studies focused on smokers [28], [36]–[40]. However, there have been controversial results or no association between the *hOGG1* 326Ser/Cys genotype and lung adenocarcinoma reported in other studies [41]–[45]. The reason for these different results between studies is not clear, but it may be because of differences in the size of the study population and differences in ethnicity.

APE1 plays a central role in base excision repair of DNA damage [46]. APE1 Asp148Glu variants are the most common APE1 polymorphisms and have been extensively studied in lung cancer; however; the results are conflicting [47]–[51]. In the current study, we found no association between APE1 Asp148Glu polymorphisms and the risk of lung adenocarcinoma among Chinese non-smoking females, which is similar to most previous studies [47]–[49]. However, an association between APE1 Asp148Glu polymorphisms and lung cancer was reported in a Japanese study [31], a Chinese study [50], and a Belgian study [51]; this might be attributed to cigarette smoking exposure. The explanation for these differences between studies is unknown, but may be due to exposure to environmental factors or joint effects with other BER genes.

ADPRT is a DNA-binding protein involved in the regulation of BER by detecting DNA strand breaks after DNA damage [52]. The association between ADPRT Val762Ala polymorphisms and lung cancer has been studied, but not extensively. A Chinese study reported that the ADPRT Ala/Ala genotype is associated with a 1.68-fold (95% CI: 1.27–2.23) increased risk with lung cancer compared with the Val/Val genotype [32]. However, no significant associations of ADPRT Val762Ala polymorphisms with lung cancer were reported in a Korean and Japanese population [33], [53]. We also did not find any significant association between ADPRT Val762Ala and the risk of lung adenocarcinoma among Chinese non-smoking females. However, the mechanisms for the effect of ADPRT Val762Ala polymorphisms on susceptibility to lung cancer remain unknown and require further investigation.

Many factors affect DNA repair activity and the risk of lung cancer, including multiple genetic variants [8]. An increasing number of studies on the joint effects of more than one variant allele were showed that complex gene-gene interactions may significantly contribute to cancer susceptibility [54], [55]. Our results suggested that a potential combined effect among homozygous genotypes of *hOGG1* 326 Cys/Cys, APE1 148Glu/Glu and ADPRT 762Ala/Ala significantly increased the risk of lung adenocarcinoma in the Chinese non-smoking female population. Therefore, individuals with more than one homozygous genotype may have a higher risk for lung adenocarcinoma.

Although tobacco smoking is the main cause of lung cancer, cancer is a multifactorial disease. Some studies have suggested that cooking oil fumes may be an environmental risk factor in lung cancer in women who do not smoke [56]–[60]. It is accepted that in addition to the individual contributions of genetic differences and environmental factors, interactions between the two are important in disease development [61]. To evaluate potential gene-environment associations, the interaction or joint effect of *hOGG1* Ser326Cys polymorphisms and cooking oil fumes on lung cancer was examined in the previous study and evidence supports an interaction between the Cys326 *hOGG1* allele and environmental exposure to ROS resulting in increased risk of cancer [62], [63]. Based on the Chinese main traditional cooking styles, including stir-frying, decoction and deep-frying, Chinese women may inhale cooking oil fumes that contain multiple potential carcinogens during prepared food. In the present study, we found that exposure to cooking oil fumes may be an environmental risk of developing lung adenocarcinoma, which is consistent with our previous study [58]–[60]. In addition, our results reflected the potential gene-environment interactions between Cys/Cys genotypes of *hOGG1* codon 326 and cooking oil fumes. This finding suggests that carrying Cys/Cys genotypes of *hOGG1* may increase the risk of lung adenocarcinoma when exposed to cooking oil fumes. Although the precise mechanism of how cooking oil fumes increase the risk in lung cancer is unclear, some studies have suggested that cooking oil fumes induce oxidative DNA damage, such as DNA adducts that are involved in lung carcinogenesis in female non-smokers and additionally, heated cooking oil increases the amount of 8-OHdG in human lung adenocarcinoma CL-3 cells, and it may directly or indirectly cause an accumulation of 8-OHdG [64], [65]. Therefore deletion, polymorphism and loss of heterozygosis of *hOGG1* could affect the overall efficiency of oxidative base damage repair, resulting in hyper-mutation phenotypes including cancer [66]. Therefore, *hOGG1* may play an important role in repairing cooking oil fume-induced DNA damage [67].

There are several limitations in the current study. First, hospital-based studies are likely to include some selection bias in the choice of controls that may not have provided a good representation of general population that alter the conclusions. Further studies with larger population-based studies are needed to reduce bias degree. Second, the statistical power of the study may be limited by the relatively small number of subjects and the number of studied SNPs, in addition, confounding factors by known and unknown risk factors could play a role in lung cancer risk and studies on the other BER genes are needed to confirm our findings in order to examine the possible relationship between BER genes polymorphisms and lung adenocarcinoma risk. Last, our study was limited to Chinese women, and the results cannot be extrapolated to other race populations. However the current study is one of the largest studies to investigate the association between BER gene polymorphisms and the risk of lung adenocarcinoma, and to evaluate the gene-gene and gene-environment interaction in the development of lung adenocarcinoma among female non-smokers. While the exact biological mechanism for the gene-environment interaction with BER gene polymorphisms is not well known, larger studies in non-smoker female populations are required in the future.

In conclusion, this hospital-based case-control study showed that *hOGG1* Ser326Cys polymorphisms might be associated with the risk of lung adenocarcinoma in Chinese non-smoking females. Furthermore, there is a significant gene-environment association between cooking oil fumes and the *hOGG1* Cys/Cys polymorphism on lung adenocarcinoma among female non-smokers.
